# Exploratory Machine Learning Analysis of circRNA-Derived Molecular Features in Autism Spectrum Disorder

**DOI:** 10.3390/ncrna12030017

**Published:** 2026-05-15

**Authors:** Raunak Sharda, Valentina L. Kouznetsova, Igor F. Tsigelny

**Affiliations:** 1 BiAna Institute, La Jolla, CA 92038, USA; raunaksharda007@gmail.com (R.S.); or vkouznetsova@ucsd.edu (V.L.K.); 2 San Diego Supercomputer Center, University of California San Diego, 9500 Gilman Dr., La Jolla, CA 92023, USA; 3 Department of Neurosciences, University of California San Diego, 9500 Gilman Dr., La Jolla, CA 92023, USA

**Keywords:** ASD, diagnostics, circRNA, machine learning

## Abstract

**Background/Objectives:** Autism Spectrum Disorder (ASD) is a set of neurological and neurodevelopmental disorders characterized by difficulties in social communication and interaction, repetitive behaviors, and sensory processing differences. Recent studies have shown that circRNAs play a crucial role in the pathophysiology of ASD. In this study, we present an exploratory machine learning framework integrating circRNA sequence features, miRNA interactions, gene targets, and pathway enrichment analysis to investigate ASD-associated molecular signatures. **Methods:** Differential circRNAs were identified from human peripheral blood datasets, and informative features were selected using attribute-based filtering and Information Gain ranking. Machine learning models were developed using the WEKA platform. **Results:** The HyperPipes classifier achieved the highest performance (92.5% accuracy under cross-validation). Analysis using an independent ASD gene expression dataset showed consistent discriminative patterns of the derived gene-level signatures across multiple machine learning classifiers. The competitive endogenous RNA network and enriched gene pathways were also analyzed. **Conclusions:** Overall, this study provides a computational, preliminary framework for analyzing circRNA-associated molecular patterns in ASD. Findings should be interpreted in the context of limited sample size and dataset availability.

## 1. Introduction

### 1.1. Background

Autism Spectrum Disorders, also known as ASDs, are a set of neurological developmental disorders that are commonly present in early childhood and are characterized by restricted social interaction, limited communication, and repetitive behaviors [1]. In the past few years, ASD occurrences have increased; these are not bound by race, ethnicity, or geographical location [2]. Currently, the diagnosis of ASD is based on behavioral observations due to a lack of biomarkers. Non-coding RNAs (ncRNAs) do not encode proteins but are often considered promising biomarkers because they regulate gene expression. Key ncRNAs include long noncoding RNAs (lncRNAs), ribosomal RNAs (rRNAs), transfer RNAs (tRNAs), small nuclear RNAs (snRNAs), small nucleolar RNAs (snoRNAs), microRNAs (miRNAs), and circular RNAs (circRNAs). miRNAs are 19–25 nucleotides in length and control gene expression primarily by silencing genes [3]. circRNAs were previously dismissed as transcriptional noise in eukaryotes [4] until recently. Previous studies have demonstrated that circular RNAs (circRNAs) play crucial functional roles in various cellular processes. They function as miRNA inhibitors or sponges [5], thereby downregulating miRNA expression and upregulating gene expression.

Innovative technology and bioinformatics have enabled us to visualize the unique structure of circular RNAs (circRNAs). circRNAs are generated by a process called backsplicing. Unlike linear mRNAs, which have 5′ and 3′ poly (A) tails, the 5′ and 3′ ends of circRNAs are covalently ligated, forming a circular RNA covalently closed loop [6]. This allows the circRNA to be highly stable and resistant to degradation. Currently, there are three main groups of circRNAs: intronic circRNAs (ciRNAs), exonic circRNAs (ecircRNAs), and exon–intron circRNAs (EIciRNA) [7]. Most circRNAs originate from the exons of protein-coding genes [8]. ecircRNAs can participate in alternative splicing, a process in which internal exons are removed following a back-splicing event [9]. In addition to their structural characteristics, ecircRNAs play important regulatory roles as miRNA sponges—binding to specific miRNAs and preventing them from repressing target mRNAs. This interaction leads to increased mRNA expression and modulation of downstream gene networks. Although several studies have investigated the functions of circRNAs and their interactions with genes, the majority of circRNA-mediated regulatory mechanisms remain largely unexplored.

### 1.2. Objective

Previous studies have identified key connections between ASD and the circRNA–miRNA–mRNA regulatory axis, also known as the competitive endogenous RNA (ceRNA) network [2]. The ceRNA network describes a regulatory framework in which different RNA molecules compete for binding to shared miRNA. miRNAs themselves are well-established post-transcriptional regulators that suppress gene expression by binding to complementary sequences in the 3′ untranslated region (3′ UTR) of target mRNAs, thereby downregulating their expression [10]. circRNAs are a class of ceRNAs that modulate miRNA availability by sequestering them via miRNA response elements, thereby influencing downstream mRNA expression.

Although previous studies, including Reiisi et al. [2], have explored circRNA-related regulatory interactions in ASD, computation approaches for systematically prioritizing ASD-associated circRNAs remain limited. In particular, publicly available datasets are scarce and often small in sample size, which poses challenges for robust computational modeling and validation.

In this study, we present an exploratory machine learning framework to characterize circRNA-associated molecular features in ASD using human peripheral blood samples. The aim is to identify informative circRNA-derived signatures that may contribute to ceRNA-related regulatory mechanisms and provide computational evidence supporting potential ASD-associated molecular patterns. Given the limited availability of large-scale circRNA datasets derived from human peripheral blood, the proposed approach is intended as a preliminary computational analysis rather than a clinically validated diagnostic model.

## 2. Results

### 2.1. Overview of circRNA Dataset and Feature Space

The GSE200197 dataset was selected to investigate circRNA-associated molecular patterns in ASD. Given the limited availability of ASD circRNA datasets meeting strict inclusion criteria (case-control design, peripheral blood origin, and compatible profiling platforms), this dataset was the only one that satisfied all methodological requirements.

The circRNA expression dataset (GSE200197) consisted of four ASD and four TD control peripheral blood samples. Differential expression analysis identified 100 differentially expressed circRNAs (DEcircRNAs), which were compared against 100 randomly selected circRNAs from circBase to construct a balanced feature space for downstream analysis.

Mature circRNA sequences were retrieved and used to derive sequence-based descriptors, including length, nucleotide composition, GC content, entropy, and *k*-mer frequencies (*k* = 2, 3, 4). In addition, circRNA–miRNA interaction features were incorporated using circBank-derived predictions, resulting in an integrated feature matrix combining sequence and interaction information.

### 2.2. Machine Learning Classification Performance

The machine learning framework was implemented using the WEKA platform (version 3.9.6) and evaluated using leave-one-out cross-validation (LOOCV), with each sample used iteratively as a test instance and the remaining samples for training. This approach provides an internal estimate of generalization performance that is suitable for small-sample settings and reduces bias and data leakage.

Multiple classifiers were evaluated under this cross-validation framework using 94 selected attributes following feature selection (Section 4.5). The performance (accuracy and evaluation metrics) of ten representative algorithms—HyperPipes, WiSARD, Bayesian Logistic Regression, Naïve Bayes, Stochastic Gradient Descent (SGD), Sequential Minimal Optimization (SMO) for Support Vector Machines, Random Forest, Instance-Based k-Nearest Neighbors (IBk), Bagging, and J48 decision tree—is summarized in Table 1.

Across all tested models, HyperPipes achieved the highest LOOCV accuracy of 92.5%, a Cohen’s Kappa of 0.86 (indicating excellent agreement beyond chance), precision of 0.936, recall of 0.930, and an F1-score of 0.925 (demonstrating balanced precision and recall). These results suggest that the HyperPipes classifier performed consistently across the evaluated metrics and was identified as the best-performing model among the exploratory machine learning classifiers in this analysis.

**Figure 1:**
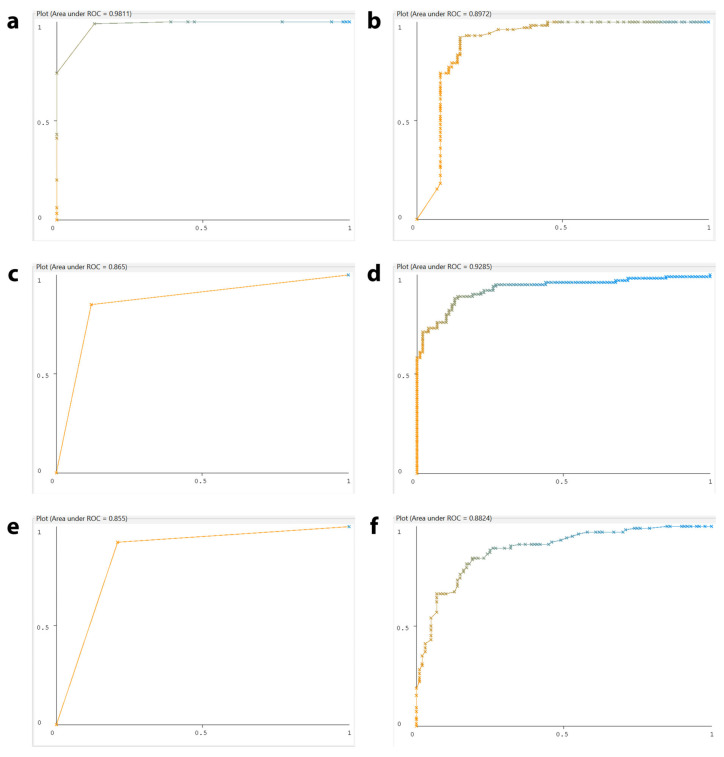

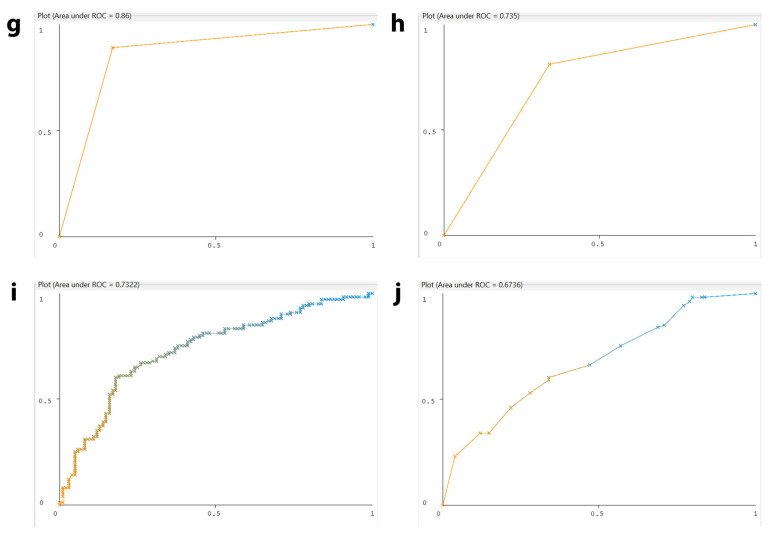
Receiver operating characteristic (ROC) curves for ten machine learning classifiers. The x-axis represents the false-positive rate, and the y-axis represents the true-positive rate. (**a**) HyperPipes classifier, AUC-ROC = 0.9784; (**b**) WiSARD classifier, AUC-ROC = 0.8972; (**c**) Bayesian Logistic Regression classifier, AUC-ROC = 0.865; (**d**) Naïve Bayes classifier, AUC-ROC = 0.9285; (**e**) SGD classifier, AUC-ROC = 0.855; (**f**) RandomForest classifier, AUC-ROC = 0.8824; (**g**) SMO classifier, AUC-ROC = 0.86; (**h**) IBk classifier, AUC-ROC = 0.735; (**i**) J48 Decision Tree classifier, AUC-ROC = 0.7322; (**j**) Bagging classifier, AUC-ROC = 0.6736.

To further evaluate and compare the discriminative performance of the machine learning models, receiver operating characteristic (ROC) curves were generated for each classifier (Figure 1). The ROC curve is a powerful tool for illustrating the relationship between the true-positive rate (TPR) and false-positive rate (FPR) across different classification thresholds. A model with a ROC curve that rises sharply toward the upper-left corner demonstrates superior performance, as it maximizes true positives while minimizing false positives. The area under the ROC curve (AUC-ROC) summarizes this performance: values approaching 1.0 indicate excellent discriminative ability, whereas values near 0.5 suggest little to no predictive power. The HyperPipes classifier achieved an AUC-ROC of 0.9784, indicating strong class separability between DEcircRNA and random circRNA. The remaining classifiers showed lower AUC-ROC values, suggesting reduced discriminative performance. Overall, HyperPipes showed the highest performance among the evaluated classifiers under the cross-validation framework.

To further assess model performance, a small supplementary evaluation was conducted using 11 DEcircRNAs reported in a previous study by Reiisi et al. [2]. The HyperPipes model correctly classified 10 out of 11 instances (90.91% accuracy). While this result is consistent with the patterns observed during cross-validation, it should be interpreted cautiously given the dataset’s limited size and lack of independence. This supplementary analysis provides additional support for the model’s prediction consistency, but it does not constitute formal external validation and has limited generalizability.

### 2.3. Analysis of the Descriptors

**Figure 2:**
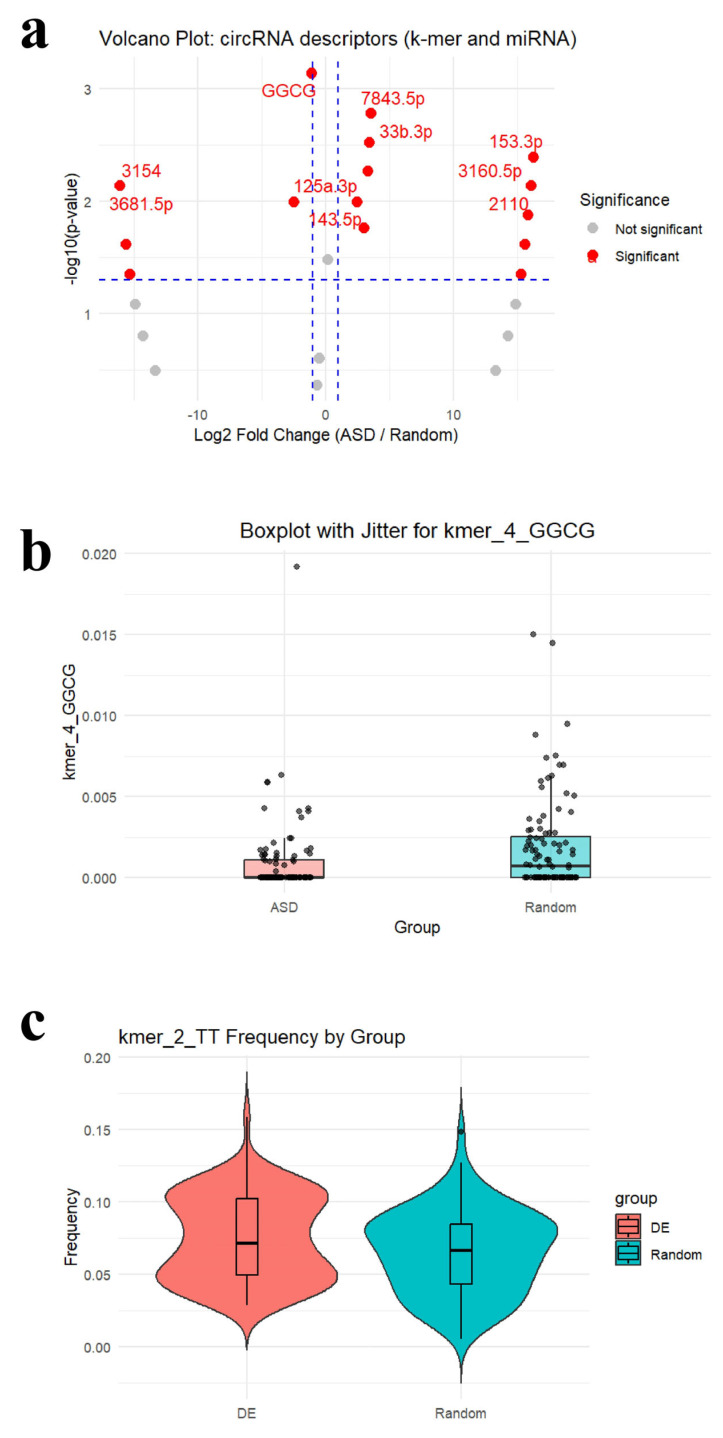
Visual Plots. (**a**) Volcano plot showing the abundance of circRNA sequence descriptors between ASD-associated circRNAs and randomly selected circRNAs. Each point represents a single descriptor, plotted by log_2_FC on the *x*-axis and −log_10_(*p*-value) from the Wilcoxon test on the *y*-axis. Features shown in red are statistically significant (*p* < 0.05 and |log_2_FC| > 1), indicating enrichment (positive *x*-axis) or depletion (negative *x*-axis) in ASD-related circRNAs. The labeled points correspond to the top 10 most significant descriptors. Certain miRNAs (e.g., 7843.5, 125a-3p) and the tetranucleotide *k*-mer descriptor GGCG exhibit large frequency differences between groups, highlighting potential relevance to ASD. The dashed lines are threshold boundaries used to separate significant biological findings from random noise. The horizontal line represents the *p*-value cutoff (statistical significance): points above the line are considered statistically significant; points below it are not. The two vertical lines represent the fold change cutoff (biological relevance): points to the right of the right line are significantly upregulated; points to the left of the left line are significantly downregulated; points between the lines represent features with little to no expression difference. (**b**) Boxplot with jitter illustrating the frequency distribution of the kmer_4_GGCG descriptor across circRNAs in the ASD and random groups. Although the median frequency is slightly lower in the ASD group, the overall distribution demonstrates significant downregulation of GGCG in ASD-related circRNAs, consistent with the volcano plot. Jittered points represent individual samples and show the spread of the descriptor, with several random circRNAs exhibiting notably higher frequencies. These findings suggest that the GGCG motif may be selectively underrepresented in ASD-associated circular RNAs, potentially reflecting an altered sequence composition. (**c**) Violin plot showing the frequency distribution of the kmer_2_TT dinucleotide motif in DEcircRNAs versus random circRNAs. DEcircRNAs display modest enrichment of this *k*-mer compared to random controls. The bimodal distribution observed in the DEcircRNA group suggests potential heterogeneity among ASD-associated circRNAs. Incorporating descriptors that capture this internal heterogeneity may improve classification accuracy by accounting for biologically meaningful variation within the ASD group.

Several exploratory visual analyses were performed to characterize sequence-derived and miRNA-derived descriptors and to highlight features that most effectively distinguish DEcircRNAs from randomly selected circRNAs (Figure 2). These analyses were conducted on the 94 descriptors retained after attribute selection, which included both *k*-mer sequence features and circRNA–miRNA interaction features. To further explore these patterns, a volcano plot, a boxplot with jitter, and a violin plot were generated to compare the distributions and statistical enrichment of key descriptors.

The volcano plot highlights the top ten statistically significant features distinguishing DEcircRNAs from random circRNAs. Features on the positive side of the *x*-axis represent enrichment in DEcircRNA, whereas those on the negative side of the *x*-axis indicate depletion in DEcircRNA. Among the enriched features, several miRNA-associated attributes—hsa-miR-7843-5p, hsa-miR-33b-3p, hsa-miR-153-3p, hsa-miR-3160-5p, hsa-miR-143-5p, and hsa-miR-2110—were upregulated in DEcircRNA, as they showed a positive log_2_ fold change and a *p*-value < 0.05, indicating higher representation in ASD-related circRNAs. In contrast, sequence-based descriptors such as kmer_4_GGCG, along with target miRNAs including hsa-miR-125a-3p, hsa-miR-3681-5p, and hsa-miR-3154, were downregulated in DEcircRNA, due to exhibiting a negative log_2_ fold change and a *p* < 0.05, suggesting their reduced abundance in DEcircRNAs.

The jittered boxplot shows a pronounced difference between ASD-related and random circRNAs in the kmer_4_GGCG sequence descriptor. DEcircRNAs display lower and more tightly clustered frequencies, whereas random circRNAs exhibit a broader range with higher outliers. This underrepresentation suggests that GGCG *k*-mer may play a regulatory role in typical circRNA function that is disrupted or absent in ASD. The lower median value observed in the ASD group further supports the hypothesis that loss of this sequence feature may be associated with ASD-related circRNAs.

The violin plot depicts the distribution of the kmer_2_TT frequencies between DEcircRNA and random circRNA. On average, DEcircRNAs show higher frequencies, suggesting potential enrichment of this dinucleotide sequence in ASD-related circRNAs. Although there is notable overlap between the two groups, the density distribution reveals distinct modes: random circRNAs peak near a frequency of 0.09, while DEcircRNAs display bimodal peaks at approximately 0.05 and 0.1. This pattern indicates heterogeneity within the DEcircRNA group, suggesting the presence of subpopulations with distinct sequence characteristics. The enrichment of specific *k*-mers, such as kmer_2_TT, in DEcircRNAs may be related to differences in structure, stability, and interactions with target miRNAs. Consequently, this sequence descriptor may provide valuable insight into understanding the molecular functions of DEcircRNAs compared to those of random circRNAs.

### 2.4. ceRNA Network

To further examine feature importance, descriptors were ranked using Information Gain as implemented in the WEKA environment. Information Gain quantifies the contribution of each attribute to class discrimination by measuring the reduction in entropy when the class label is known. This approach was applied to the subset of 94 descriptors retained after application of *Attribute Selection* function, allowing identification of the most informative features.

The top five highest ranking descriptors based on Information Gain are presented in Table 2. These features included both sequence-derived *k*-mers and predicted miRNA interaction features, indicating that both sequence composition and miRNA targeting patterns contribute to distinguishing DEcircRNAs from randomly selected circRNAs.

**Figure 3:**
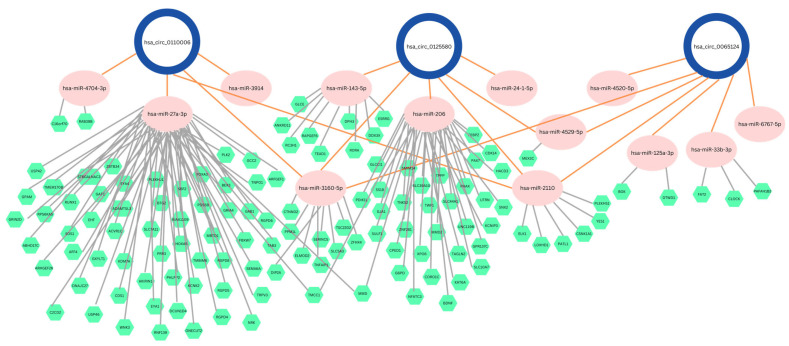
ceRNA network for hsa-circ-0110006, hsa-circ-0125580, and hsa-circ-0065124. A competing endogenous RNA (ceRNA) network was constructed for the three selected circular RNAs (circRNAs). Blue circles represent the circRNAs, pink ovals denote their target miRNAs, and green hexagons indicate their target genes. Orange edges connect each circRNA to its corresponding target miRNAs, while grey edges connect the miRNAs to their predicted target genes.

To further contextualize these findings within a regulatory framework, a competing endogenous RNA (ceRNA) network was constructed using three influential DEcircRNAs (Figure 3). This schematic illustrates predicted interactions between circRNAs, miRNAs, and downstream gene targets. The gene targets were collected from the miRDB database using a strict threshold of >97. In total, 13 miRNAs were identified as interacting with one or more of the selected circRNAs, connecting to 124 predicted gene targets. Among these, hsa-miR-2110 was associated with all three circRNAs, suggesting a potentially central role within the constructed network. This network representation is intended to provide a conceptual view of potential regulatory relationships rather than definitive functional validation.

### 2.5. Gene Collection and Validation

To further investigate the regulatory landscape of ASD-related circRNAs, genes were collected based on the 90 most significant miRNAs from the *Attribute Selection* filter. Using the miRDB database with a target accuracy score threshold of 97, 693 genes were retrieved. Collecting these genes is important because they provide additional insight into the potential ceRNA network underlying ASD pathology.

To assess the biological relevance of this gene set, an independent peripheral blood gene expression dataset (GSE18123) was used as a reference. After preprocessing and mapping, 664 of the 693 predicted genes (95.8%) overlapped with genes measured in the dataset and were retained for further analysis. This step ensured consistency between computationally predicted targets and experimentally observed gene expression profiles.

Rather than serving as a formal external validation of the predictive model, this analysis was designed to evaluate whether the identified gene set exhibits a discriminatory signal in an independent ASD-related dataset and is therefore biologically relevant to ASD. To this end, multiple standard classifiers (Random Forest, Support Vector Machine, and Logistic Regression) were applied using 10-fold cross-validation. These models achieved moderate and consistent classification performance (accuracies ranging from 71.9% to 79.7%), indicating that the gene set captures patterns associated with ASD in an external dataset.

Given differences in data origin, platform, and study design, this step should be interpreted as a supportive functional assessment of the predicted gene set, rather than definitive validation. Nonetheless, the observed consistency across classifiers suggests that the identified genes may reflect biologically relevant components of ASD-associated regulatory mechanisms.

### 2.6. Pathway Enrichment Analysis

Pathway enrichment analysis was performed using two complementary resources:

- The KEGG (Kyoto Encyclopedia of Genes and Genomes platform (www.genome.jp/kegg, accessed on 6 December 2025) [11] via the DAVID platform (davidbioinformatics.nih.gov, accessed on 8 December 2025) [12].
- Reactome (reactome.org, accessed on 19 December 2025) [13], a comprehensive resource for enriched biological pathways, used to analyze the list of significant genes for biological relevance.

The top 25 enriched functional pathways identified in the KEGG Functional Annotation Chart and Reactome Pathway Analysis Results are summarized in Table 3.

### 2.7. KEGG Pathway Enrichment

KEGG analysis of the elucidated important genes revealed significant enrichment in several signaling pathways known to influence neural development and synaptic function, including the ErbB, Neurotrophin, and PI3K–Akt pathways.

*ErbB signaling pathway*: Regulates cell proliferation, differentiation, survival, and migration. It is essential for brain development and the formation of neural circuits.

*Neurotrophin signaling pathway*: Supports neuronal differentiation and growth, regulates axon guidance, and facilitates synaptic plasticity, which is crucial for learning and memory.

*PI3K–Akt signaling pathway*: Promotes cell survival by inhibiting apoptosis and contributes to neuronal development and synaptic plasticity.

Additionally, enrichment of proteoglycans in the cancer pathway suggests involvement of genes that influence neural development, as proteoglycans are key components of the neuronal extracellular matrix and play important roles in axon guidance, synaptic organization, and neural plasticity. Dysregulation of proteoglycan-mediated signaling may therefore be biologically relevant to ASD and useful for identifying ASD-related genes. The enrichment of this pathway, along with other signaling pathways, provides important insights into the molecular mechanisms underlying ASD, where disruptions in neural signaling are widely recognized as contributing to its pathogenesis.

### 2.8. Reactome Pathway Enrichment

Reactome analysis also revealed strong involvement of pathways related to signal transduction and gene expression regulation, including those involving key transcription factors and growth factor-mediated signaling processes essential for neural development. Reactome pathways ranked 7, 8, 11, 13, and 14 featured multiple transcription factors (MECP2, RUNX1, RUNX2, YAP1/TAZ), which are critical regulators of neuronal differentiation and maturation. The repeated appearance of *MECP2*, an essential central epigenetic regulator in human brain development, whose gene mutations are often associated with Rett syndrome [14], underscores its importance in the context of ASD-related molecular pathways. Additional pathways, such as BMP signaling and YAP1/TAZ-stimulated gene expression, both of which transmit extracellular signals that activate transcription, highlight the functional link between circRNA-associated genes and developmental signaling cascades.

### 2.9. Convergence of KEGG and Reactome Pathways

Substantial overlap was observed between KEGG and Reactome pathway enrichment results, with several biological pathways and signaling networks shared between the two. The enrichment of the ErbB signaling pathway (KEGG Rank 1) is similarly projected by the Reactome Downregulation of ERBB4 signaling (Reactome Rank 12) and signaling by ERBB2 KD Mutants (Reactome Rank 24) pathways.

Enrichment in the Neurotrophin signaling KEGG pathway (KEGG Rank 4) aligns with p75NTR recruiting signaling complexes (Reactome Rank 25) because the p75NTR neurotrophic receptor is fundamental to the role of neurotrophins in modulating brain plasticity and apoptosis [15].

The TGF-β signaling KEGG pathway (KEGG Rank 7) directly corresponds to Reactome signaling by the TGF-β family (Reactome Rank 15). The TGF-β pathway is crucial to the cellular signaling system that regulates cell growth, differentiation, apoptosis, and immune response. The presence of this pathway in both KEGG and Reactome analyses demonstrates its high biological relevance and strengthens confidence in its potential role in ASD. The convergence of multiple key signaling pathways across both databases provides robust evidence for their involvement in gene regulation related to ASD.

## 3. Discussion

Autism spectrum disorder (ASD) significantly impacts the patient’s quality of life and continues to rise in incidence worldwide [16]. Despite extensive research over recent years, the underlying pathogenesis of autism remains unclear [2]. Recent advances in circRNA biology, bioinformatics, and machine learning have opened new directions for investigating ASD biomarkers and molecular mechanisms.

In this study, we developed an exploratory ML framework integrating circRNA sequence-derived features and predicted circRNA–miRNA interactions derived from human peripheral blood samples. Using a small yet carefully selected dataset, 94 informative descriptors were identified that strongly distinguish DEcircRNAs from randomly selected circRNAs. These features included both sequence-based *k*-mer patterns and miRNA interaction profiles, supporting the idea that both structural and regulatory characteristics contribute to circRNA-associated variation in ASD.

The classification analysis demonstrated that multiple ML algorithms could distinguish between circRNA groups under an LOOCV framework, with HyperPipes achieving the highest overall performance across the evaluated metrics. However, given the limited sample size, these results should be interpreted cautiously. The discrepancy between high training performance and more moderate cross-validation results suggests potential sensitivity to overfitting, despite the use of LOOCV and feature selection to mitigate this risk. The supplementary evaluation using a small, literature-derived circRNA set provided additional, albeit limited, support for pattern consistency, but does not represent independent validation.

Predicted miRNA targets were used to derive a downstream gene set, enabling construction of a putative circRNA–miRNA–mRNA regulatory network. When evaluated in an independent peripheral blood gene expression dataset, this gene set demonstrated moderate but consistent classification performance across multiple standard classifiers. This step was not intended as a validation of the original model, but rather as a functional assessment to determine whether the identified gene signatures capture ASD-related signals in an external dataset. The results suggest that the predicted targets may reflect biologically relevant processes, although differences in dataset origin, platform, and study design limit direct comparability.

Pathway enrichment analysis of the gene set identified several signaling pathways involved in transcriptional regulation, cell signaling, and neurodevelopmental processes. The involvement of specific pathways, such as BMP signaling, YAP/TAZ signaling, and MECP2-regulated transcription, shows a bridge between extracellular signaling and gene expression control. These findings are consistent with earlier work [17], reinforcing the central role of disrupted signal transduction of molecular events involved in pathological processes such as transcription and translation. Given that the underlying data were derived from peripheral blood rather than neural tissue, these findings should be interpreted cautiously, as they may reflect systemic regulatory signals rather than direct central nervous system mechanisms.

Despite these promising results, this study has some important limitations. The availability of circRNA expression datasets derived from peripheral blood samples in ASD remains limited, restricting this analysis to a single small cohort that may not fully capture the heterogeneity of the broader ASD population. This constraint also increases the risk of overfitting; however, measures such as LOOCV, feature selection, and evaluation metrics were used to provide a more conservative assessment of model performance. The absence of comparable circRNA datasets further prevented independent evaluation under similar conditions, although a gene-level analysis using an external peripheral blood dataset provided indirect but supportive evidence for the biological relevance of the identified signatures. Nonetheless, differences in data sources and platforms limit direct comparability. Additionally, this study relies entirely on computational predictions without experimental validation of the proposed circRNA–miRNA–gene interactions. Future studies incorporating larger, more diverse datasets and experimental validation will be essential to improve robustness, generalizability, and biological interpretation. They will also further clarify the role of circRNA-associated regulatory mechanisms in ASD.

Taken together, this work provides a preliminary computational framework for integrating circRNA sequence features, miRNA interactions, and downstream gene analysis in ASD. These findings highlight potentially informative molecular patterns that may contribute to ceRNA-related regulatory mechanisms. Future studies incorporating larger cohorts and experimental validation will be necessary to confirm these observations and determine their biological and clinical relevance.

## 4. Materials and Methods

### 4.1. Microarray Data Collection

To obtain circRNA expression datasets relevant to autism spectrum disorder (ASD), a systematic search was conducted in the Gene Expression Omnibus (GEO) database (NCBI; Bethesda, MD, USA) [18]. The search strategy focused on identifying studies that included (i) ASD and typical development (TD) control samples, (ii) circRNA expression profiling data, (iii) derivation from human peripheral blood samples, and (iv) consistent experimental platforms suitable for comparative analysis. This structured screening process ensured that only datasets that met the minimum biological and technical compatibility criteria were considered.

Based on this search strategy, dataset GSE200197 (NCBI; Bethesda, MD, USA) [19] was selected. The dataset was generated using the Agilent-084217 CapitalBio Technology Human CircRNA Array v2 (platform GPL28148) (CapitalBio Technology Co., Ltd., Beijing, China) [20]. The extracted data contained four ASD samples and four TD control samples derived from human peripheral blood.

### 4.2. Dataset Justification and Study Constraints

The selection of GSE200197 was based on strict inclusion criteria; however, it is important to note that publicly available circRNA datasets for ASD that include both case–control design and peripheral blood-derived samples are extremely limited. Despite an extensive search, no additional GEO datasets met all required conditions simultaneously, particularly for circRNA profiling and peripheral blood samples.

As a result, GSE200197 represents the only dataset that fulfills the study’s methodological requirements, and no additional datasets were available for independent external validation under comparable conditions. This reflects a broader limitation in the field, where circRNA-based ASD datasets remain scarce and underdeveloped.

To address these constraints and the limited sample size of the GSE200197 dataset, model performance was evaluated using leave-one-out cross-validation (LOOCV), where each sample was used as a test instance, and the remaining samples were used for training. To partially support robustness, supplementary evaluation was performed using a literature-derived small set of ASD-associated circRNAs, although this does not constitute a fully independent validation cohort. Given these constraints, the proposed machine learning framework should be interpreted as an exploratory model aimed at identifying potential circRNA-associated patterns in ASD rather than a clinically validated diagnostic tool.

### 4.3. Identification of DEcircRNA

We utilized the GEO2R interactive web tool, part of the NCBI Gene Expression Omnibus (GEO) database [21], on the dataset GSE200197. Log-fold change (logFC) values and *p*-values were calculated using the Benjamin–Hochberg (BH) false discovery rate correction method [22]. circRNAs with a *p*-value < 0.05 and |logFC| > 2.0 were considered statistically significant and differentially expressed (DE). The top 100 DEcircRNAs identified by GEO2R were selected for subsequent analyses. For comparison, 100 circRNAs were obtained from the circBase database (Rajewsky Lab at the Berlin Institute for Medical Systems Biology, Max Delbrück Center for Molecular Medicine, Berlin, Germany; https://www.circbase.org/, accessed on 15 October 2025) [23], selected using a random number generator without repetition. Any circRNAs that overlapped with the DEcircRNA set were excluded and replaced to ensure two distinct groups.

### 4.4. circRNA Descriptors

To obtain mature circRNA sequences, we utilized the circBank database (ATCGene Inc., Guangzhou, China, in collaboration with the Sunnybrook Research Institute, University of Toronto, Toronto, ON, Canada; https://www.circbank.cn, accessed on 17 October 2025) for each circRNA [24]. The length, GC Content, Entropy, and nucleotide composition were collected as features. Furthermore, *k*-mer features were also generated based on the nucleotide combinations present in each sequence. These features represent the frequencies of all subsequences of length *k*, providing insights into the underlying sequence composition of each circRNA. Specifically, we calculated *k*-mer frequencies for dinucleotides (*k* = 2), trinucleotides (*k* = 3), and tetranucleotides (*k* = 4) across each mature circRNA sequence. This approach quantifies the occurrence of each nucleotide, showcasing the underlying sequence structure of each circRNA. It captures both simple and complex sequence patterns that may play important roles in circRNA structure and regulation. The resulting *k*-mer descriptors served as important informative sequence-based features for downstream analysis.

In addition, we used the circBank database to collect and retrieve the predicted miRNA targets of the specified circRNAs. This database employs a unique scoring system that indicates the confidence level of each predicted circRNA–miRNA interaction. We selected target miRNAs with a strict threshold of ≥150 in order to obtain statistically significant miRNAs as features. This resulted in 2453 target miRNA features, which were organized into a binary interaction matrix.

### 4.5. Data Preprocessing

Given the high dimensionality of the dataset and relatively smaller number of samples, feature selection was a critical step to eliminate noisy variables that could negatively impact model generalization and interpretability. Due to the large number of features, dimensionality reduction was necessary to mitigate overfitting and improve computational efficiency.

Attribute selection was performed using the Waikato Environment for Knowledge Analysis (WEKA), version 3.9.6 (University of Waikato, Hamilton, New Zealand) [25,26] as a preprocessing step to reduce dimensionality prior to model development and evaluation. The procedure was applied to the full dataset to identify the most informative descriptors using *CfsSubsetEval* function in WEKA, which selects feature subsets based on their correlation with class label while minimizing redundancy between features. To further assess individual feature relevance, Information Gain was also used as a complementary ranking criterion.

This procedure identified 94 informative descriptors, including both sequence-derived *k*-mer features and circRNA–miRNA interaction features. These selected features were subsequently used as input variables for model evaluation via leave-one-out cross-validation (LOOCV) with 8 folds.

### 4.6. ML Model Training and Validation

Machine learning models were developed and evaluated using the Waikato Environment for Knowledge Analysis (WEKA), version 3.9.6 [25]. Given the limited dataset size, a leave-one-out cross-validation (LOOCV) strategy was employed to maximize the use of available data while providing an internal estimate of model generalization performance. In this approach, each sample was used sequentially as a test instance, while the remaining samples were used for model training. This procedure is equivalent to *k*-fold cross-validation, where *k* equals the total number of samples, and is commonly adopted in small-sample bioinformatics studies to reduce bias in performance estimation.

Multiple classification algorithms were evaluated, and performance was assessed within the WEKA environment. In addition to overall accuracy, multiple complementary performance metrics were used to provide a more comprehensive evaluation of model performance, including Cohen’s kappa, F1-score, precision, recall, specificity, area under the receiver operating characteristic curve (AUC-ROC), and confusion matrix-based analysis. This multi-metric evaluation was conducted to ensure a more robust assessment of classifier performance, particularly given the limited sample size and class imbalance.

To further assess model performance, a supplementary evaluation was conducted using a set of 11 differentially expressed circRNAs identified in a previously published study [2] and associated with ASD peripheral blood samples. This literature-derived set was not used for model training or feature selection and was applied as an external reference for additional performance checks rather than for formal validation. HyperPipes showed consistent predictive performance on this dataset, achieving an accuracy of 90.91%, supporting the stability of the model’s learned patterns across related circRNA signatures. It is important to note that this literature-derived set is small and does not represent a fully independent or large-scale validation cohort. Therefore, while this supplementary analysis provides supportive evidence of pattern consistency, it remains limited in statistical strength and generalizability.

### 4.7. Feature Analysis

Information Gain (InfoGain) was first used to rank features based on their ability to discriminate between DEcircRNAs and randomly selected circRNAs. InfoGain was applied in the WEKA environment as part of the feature evaluation process to quantify the relevance of each descriptor to the class label. The resulting ranking was used to identify the most informative sequence-derived and miRNA-related features.

To further examine feature distributions, we performed visualization-based analyses using volcano plots, jittered boxplots, and violin plots. These visualizations were used to highlight similarities and differences in *k*-mer and miRNA-related descriptors.

A competing endogenous RNA (ceRNA) network was constructed using the three highest-ranked DEcircRNAs, ranked by Information Gain scores. Predicted circRNA–miRNA interactions were retrieved, followed by the identification of downstream gene targets. Gene targets were obtained using the miRDB database, with a target score threshold of >97 applied to retain high-confidence interactions. These interactions were integrated to construct a circRNA–miRNA–gene regulatory network representing potential ceRNA relationships. The final network was visualized to illustrate interaction patterns among circRNAs, miRNAs, and target genes.

### 4.8. Gene Collection and Functional Validation

Gene targets associated with the 90 statistically significant miRNAs identified through the *Attribute Selection* filter were retrieved using the miRDB database [27]. Target prediction was performed using a confidence threshold of > 97 to ensure high-confidence interactions, yielding 693 unique gene targets. These genes were subsequently used for downstream validation and functional assessment.

For external validation of the gene set, the GEO dataset GSE18123 (NCBI; Bethesda, MD, USA) was used. This dataset contains peripheral blood gene expression profiles generated using the Affymetrix Human Genome U133 Plus 2.0 Array (GPL570) platform (Affymetrix, Inc., Santa Clara, CA, USA), including ASD and control samples. For this study, a subset of 31 ASD and 33 control samples was selected to validate the original gene targets. This dataset was selected because it represents one of the largest publicly available peripheral blood transcriptome datasets for ASD and has been previously used for classification-based gene expression studies.

Gene expression data from GSE18123 were processed to match the gene list derived from miRNA target prediction. Of the 693 genes identified from miRDB analysis, 664 genes overlapped with the GSE18123 expression dataset (95.8%), and only these overlapping genes were retained for downstream validation. This ensured consistency between predicted targets and experimentally measured gene expression profiles.

To evaluate whether the identified gene set was related to ASD and could discriminate between ASD and control samples, multiple machine learning classifiers were applied, including Random Forest, Support Vector Machine (SVM), and Logistic Regression. Using 10-fold cross-validation, the classifiers achieved accuracies of 71.9% (Random Forest), 79.7% (SVM), and 73.4% (Logistic Regression), demonstrating consistent classification performance across different algorithms.

This external validation step was used to assess the biological relevance and discriminative potential of the predicted gene set rather than to develop a primary diagnostic model. Given the dataset’s retrospective nature and differences in experimental sources, results should be interpreted as supportive functional evidence rather than definitive validation.

### 4.9. Gene Pathway Analysis

Functional enrichment analysis was performed using the final set of 664 genes to investigate their involvement in biological processes and signaling pathways. Kyoto Encyclopedia of Genes and Genomes (KEGG; Kanehisa Laboratories at the Kyoto University Bioinformatics Center, Kyoto, Japan) [11] and Reactome (Ontario Institute for Cancer Research, Toronto, ON, Canada; NYU Langone Health, New York, NY, USA; Oregon Health & Science University, Portland, OR, USA; and European Bioinformatics Institute, Hinxton, UK) [13] pathway analyses were conducted using the Database for Annotation, Visualization, and Integrated Discovery (DAVID) software, v2025_2 (Laboratory of Human Retrovirology and Immunoinformatics (LHRI), Frederick National Laboratory for Cancer Research, Frederick, MD, USA) [12]. This analysis was performed to identify enriched molecular pathways and better understand the biological mechanisms and functional relationships associated with ASD-related gene signatures derived from the circRNA–miRNA regulatory network.

## Figures and Tables

**Table 1:** Performance Accuracy and Evaluation Metrics of Classifiers.

Classifier	Accuracy (%)	Cohen’s Kappa	Precision	Recall	F1-Score	Confusion Matrix (TP/FN/FP/TN) ^†^
HyperPipes	92.5	0.86	0.936	0.930	0.930	99/1/13/87
WiSARD	90.5	0.81	0.906	0.905	0.905	93/7/12/88
Bayesian Logistic Regression	86.5	0.73	0.865	0.865	0.865	85/15/12/88
Naïve Bayes	86.5	0.73	0.866	0.865	0.865	89/11/16/84
SVM	86	0.72	0.861	0.860	0.860	89/11/17/83
SGD	85.5	0.71	0.861	0.855	0.854	92/8/21/79
Random Forest	81.5	0.63	0.816	0.815	0.815	84/16/21/79
IBk	73.5	0.47	0.740	0.735	0.734	81/19/34/66
Bagging	70	0.40	0.702	0.700	0.699	65/35/25/75
J48	63	0.26	0.630	0.630	0.630	60/40/34/66

^†^ TP = True Positive; FN = False Negative; FP = False Positive; TN = True Negative.

**Table 2:** The top five strongest descriptors.

Descriptor	Info Gain Score
kmer_4_CCCG	0.1102
kmer_2_TT	0.0627
hsa-miR-153-3p (target)	0.0412
hsa-miR-2110 (target)	0.0359
hsa-miR-3154 (target)	0.0359

**Table 3:** Top 25 Enriched Functional Pathways.

Rank (KEGG)	KEGG Pathway	*p*-Value	Rank (Reactome)	Reactome Pathway	*p*-Value
1	ErbB signaling pathway ^†^	0.00026	1	Transcriptional regulation by MECP2	0.0000171
2	Prostate cancer	0.00081	2	RUNX1 regulates estrogen receptor-mediated transcription^†^	0.000167
3	Proteoglycans in cancer	0.0012	3	Downregulation of SMAD2/3:SMAD4 transcriptional activity	0.000753
4	Neurotrophin signaling pathway	0.0013	4	Activation of RAC1 ^†^	0.001032
5	PI3K–Akt signaling pathway ^†^	0.0015	5	Specification of the neural plate border	0.001446
6	Longevity-regulating pathway	0.0015	6	Signaling by BMP	0.00152
7	TGF-β signaling pathway ^†^	0.0018	7	MECP2 regulates transcription factors	0.001711
8	Human immunodeficiency virus 1 infection	0.0019	8	Transcriptional regulation by RUNX1	0.001735
9	Human cytomegalovirus infection	0.0036	9	Diseases of signal transduction by growth factor receptors and second messengers	0.001835
10	Regulation of actin cytoskeleton ^†^	0.0046	10	Regulation of MITF-M-dependent genes involved in apoptosis	0.002152
11	Spinocerebellar	0.0061	11	*YAP1*- and *WWTR1* (*TAZ*)-stimulated gene expression ^†^	0.002276
12	Hepatitis B	0.0063	12	Downregulation of *ERBB4* signaling ^†^	0.002409
13	Axon guidance	0.0071	13	*RUNX2* regulates genes involved in the differentiation of myeloid cells	0.003539
14	Shigellosis	0.0096	14	*MECP2* regulates transcription of genes involved in GABA signaling	0.003539
15	Choline metabolism in cancer	0.0097	15	Signaling by TGFB family members^†^	0.00431
16	Human papillomavirus infection	0.0097	16	*MECP2* regulates transcription of neuronal ligands	0.004348
17	Yersinia infection	0.012	17	NOD1/2 Signaling Pathway	0.004966
18	Hippo signaling pathway ^†^	0.012	18	Downstream signaling of activated FGFR1	0.005638
19	T cell receptor signaling pathway	0.013	19	Ephrin signaling	0.006341
20	Focal adhesion	0.016	20	Interaction between L1 and Ankyrins	0.006744
21	PD-L1 expression and PD-1 checkpoint pathway in cancer	0.016	21	Signaling by GSK-3β mutants	0.007134
22	Focal adhesion	0.016	22	Deactivation of the β-catenin transactivating complex	0.007183
23	Breast cancer ^†^	0.019	23	MAP kinase activation^†^	0.008637
24	Endometrial cancer ^†^	0.020	24	Signaling by ERBB2 KD Mutants ^†^	0.008845
25	Gastric cancer	0.021	25	p75NTR recruits signaling complexes ^†^	0.008886

^†^ Pathways are ranked independently by *p*-value. KEGG and Reactome Pathways listed side-by-side are not necessarily matched; they are listed for comparative purposes. Indicates biologically related pathways identified in both databases, even if ranked or named differently.

## Data Availability

The data presented in this study are derived from publicly available sources. circRNA expression data were obtained from the National Center of Biotechnology Information Gene Expression omnibus (NCBI GEO) under the accession number GSE200197 (https://www.ncbi.nlm.nih.gov/geo/query/acc.cgi?acc=GSE200197, accessed on 23 October 2025). Gene expression data used for supporting evaluation were obtained from GSE18123 (https://www.ncbi.nlm.nih.gov/geo/query/acc.cgi?acc=GSE18123; accessed on 25 October 2025). Additional circRNA annotation data were obtained from circBase (https://www.circbase.org; accessed on 15 October 2025;) and circBank (https://www.circbank.cn; accessed on 17 October 2025). The external dataset consisting of 11 differentially expressed circRNAs was extracted from a previously published study [2]. All datasets are publicly available from their respective sources.
